# Urinary Volatomic Expression Pattern: Paving the Way for Identification of Potential Candidate Biosignatures for Lung Cancer

**DOI:** 10.3390/metabo12010036

**Published:** 2022-01-04

**Authors:** Khushman Taunk, Priscilla Porto-Figueira, Jorge A. M. Pereira, Ravindra Taware, Nattane Luíza da Costa, Rommel Barbosa, Srikanth Rapole, José S. Câmara

**Affiliations:** 1 Proteomics Lab, National Centre for Cell Science (NCCS), Ganeshkhind, SPPU Campus, Pune 411007, India; khushmantaunk@nccs.res.in (K.T.); ravindra.taware@niperahm.res.in (R.T.); 2 CQM—Centro de Química da Madeira, Centro de Ciências Exatas e da Engenharia, Universidade da Madeira, Campus Universitário da Penteada, 9020-105 Funchal, Portugal; priscilla.figueira@staff.uma.pt (P.P.-F.); jorge.pereira@staff.uma.pt (J.A.M.P.); 3 Instituto de Informática, Alameda Palmeiras, Quadra D, Campus Samambaia, Universidade Federal de Goiás, Goiânia 74690-900, GO, Brazil; nattane.luiza@ifgoiano.edu.br (N.L.d.C.); rommel@inf.ufg.br (R.B.); 4 Faculdade de Ciências Exatas e da Engenharia, Universidade da Madeira, Campus Universitário da Penteada, 9020-105 Funchal, Portugal

**Keywords:** lung cancer (LC) biomarkers, volatile organic metabolites (VOMs), HS-SPME, GC-qMS

## Abstract

The urinary volatomic profiling of Indian cohorts composed of 28 lung cancer (LC) patients and 27 healthy subjects (control group, CTRL) was established using headspace solid phase microextraction technique combined with gas chromatography mass spectrometry methodology as a powerful approach to identify urinary volatile organic metabolites (uVOMs) to discriminate among LC patients from CTRL. Overall, 147 VOMs of several chemistries were identified in the intervention groups—including naphthalene derivatives, phenols, and organosulphurs—augmented in the LC group. In contrast, benzene and terpenic derivatives were found to be more prevalent in the CTRL group. The volatomic data obtained were processed using advanced statistical analysis, namely partial least square discriminative analysis (PLS-DA), support vector machine (SVM), random forest (RF), and multilayer perceptron (MLP) methods. This resulted in the identification of nine uVOMs with a higher potential to discriminate LC patients from CTRL subjects. These were furan, o-cymene, furfural, linalool oxide, viridiflorene, 2-bromo-phenol, tricyclazole, 4-methyl-phenol, and 1-(4-hydroxy-3,5-di-tert-butylphenyl)-2-methyl-3-morpholinopropan-1-one. The metabolic pathway analysis of the data obtained identified several altered biochemical pathways in LC mainly affecting glycolysis/gluconeogenesis, pyruvate metabolism, and fatty acid biosynthesis. Moreover, acetate and octanoic, decanoic, and dodecanoic fatty acids were identified as the key metabolites responsible for such deregulation. Furthermore, studies involving larger cohorts of LC patients would allow us to consolidate the data obtained and challenge the potential of the uVOMs as candidate biomarkers for LC.

## 1. Introduction

**Figure 1:**
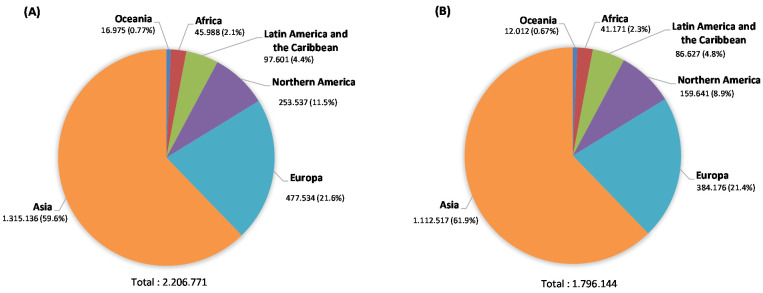
Estimated number of LC new cases in 2020 in both sexes and all ages (**A**) and estimated number of deaths (**B**) (data source: Globocan2020 [1]).

Lung cancer (LC) ranks as the second most diagnosed type of cancer worldwide and simultaneously is the leading cause of cancer deaths. The most recent data available from Globocan 2020 points to 1.8 million deaths, 18% of total cancer deaths (Figure 1), a feature that is also verified in India subcontinent [1,2]. As can be observed in Figure 1, Micronesia/Polynesia, Eastern and Southern Europe, Eastern Asia, and Western Asia are the regions sharing the highest incidence rates for LC. In contrast, incidence in the African continent is generally low, although they range from intermediate to high in both the southern and northern regions of Africa [3].

LC trends are tightly correlated with smoking and this behaviour is considered responsible for over 80% of LC cases in western populations. This fact drove broad campaigns for smoking cessation and exposure all over the world [4]. Nevertheless, LC incidence and mortality remain very high and are expected to continue to rise worldwide in the coming decades [1]. The currently used methods to diagnose LC involving tomography scans rely on tumour size, which is effective in late stages of the disease, but unsuitable for early diagnosis, when the diseases could be easily mitigated. In this point, low-dose computed tomography (LDCT) screening seems to be more effective in the identification of LC patients at early and operable stages [5]. Nevertheless, these methods are expensive, time-consuming, and involve radiation exposure hazards, and thus, they are not amenable to widespread screening [2,6]. In this context, over the last few decades, great efforts have been made to unveil highly sensitive and specific biomarkers of LC through the metabolic characterisation of different biofluids [7,8,9]. This approach is based on the fact that cancer cell metabolism is inherently different from normal cell metabolism, therefore producing metabolic biosignatures that can be used to discriminate cancer patients from healthy individuals. Different studies have shown the potential of such metabolic strategies using human biofluids—including plasma [10], serum [11,12], sweat [13], sputum [14], or urine [10,15,16]. Such metabolic studies incorporating data information from all metabolites found in human biofluids involve fewer variables than genomics and transcriptomics studies [17] but are still complex and hard to analyse [18,19]. To overcome this challenge, researchers focus their attention on subsets of the human metabolites produced by the cells as the volatile organic metabolites (VOMs). Production of VOMs and release by cells reflects their metabolic activity and thus there is obvious potential in VOMs as disease biomarkers, including LC. Accordingly, different strategies, mainly using the gold standard of solid-phase microextraction combined with gas chromatography mass spectrometry (SPME-GC/MS), has been used to identify volatile biosignatures and putative volatile LC biomarkers in human biofluids [20], particularly exhaled breath [21,22,23], pleural effusions [24], blood [25], and urine [26]. In this respect, urine has been long recognised as a valuable matrix for disease diagnosis by linking specific VOMs found in urine to medical conditions. As most VOMs are metabolised in the liver and excreted in the urine, they contain important systemic information about the clinical condition of the organisms [27]. The altered levels of aldehydes generated by lipid oxidation and membrane peroxidation, for instance, can be correlated with inflammation, necrosis, and cancer cell development [6]. Moreover, urine sampling is safe and non-invasive, easy to perform, inexpensive, and does not require any specialised medical expertise [28,29,30]. However, the potential of such an approach is hindered by several factors, such as the absence of standardisation across different studies, complex and various data analysis tools, difficulties in recruiting many subjects and obtaining many samples in controlled environments and conditions, or the existence of many interferences (genetic background, diet, unhealthy lifestyle habits, environmental contamination, etc.). Such interferences make it difficult to elucidate the metabolomic origin of many urinary VOMs [31]. Hence, research in this field still has many challenges to overcome and more extensive studies are necessary to generate reliable data to elucidate trends, biochemical routes and hopefully VOMs signatures and biomarkers which would eventually be able to allow an early LC diagnosis in future. 

In this context, Hanai et al. [32,33] reported promising results by applying HS-SPME/GC-MS to analyse the urinary volatile composition of LC cancer patients and healthy individuals. More recently, research from the Universidad de Salamanca [26,34,35] using a similar volatomic approach reported several VOMs with a great discriminative ability for LC urine samples. 

This study reports the analysis of the volatile composition of urine samples from LC patients and healthy individuals in an Indian population using HS-SPME/GC-MS. To the best of our knowledge, this is the first time such a study has been applied in this population and the results obtained reveal the potential of the application of such a strategy.

## 2. Results and Discussion

### 2.1. Characterisation of the Urinary Volatile Composition of LC Patients

The volatile composition of urine samples from LC patients and healthy volunteers, in a total of 55 subjects was analysed using the procedure described in the Materials and Methods section. The characterisation of the recruited subjects in terms of age, gender, and smoking habits is available in Table 1.

**Figure 2:**
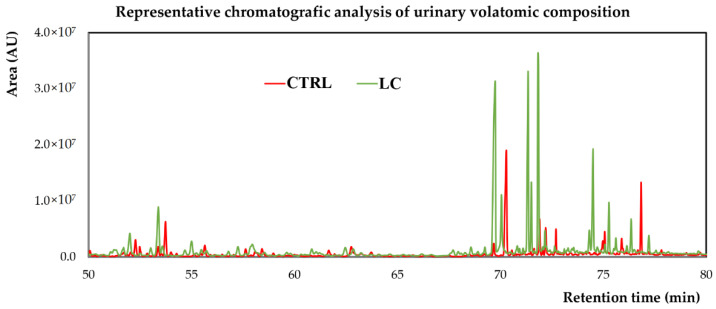
Representative chromatogram of urine sample from a control subject (CTRL) and a LC patient. AU—arbitrary units.

Figure 2 shows a snapshot of typical GC-qMS total ion chromatograms (TICs) obtained, where it is possible to observe pronounced differences in the volatomic profiles of the two groups analysed. 

**Figure 3:**
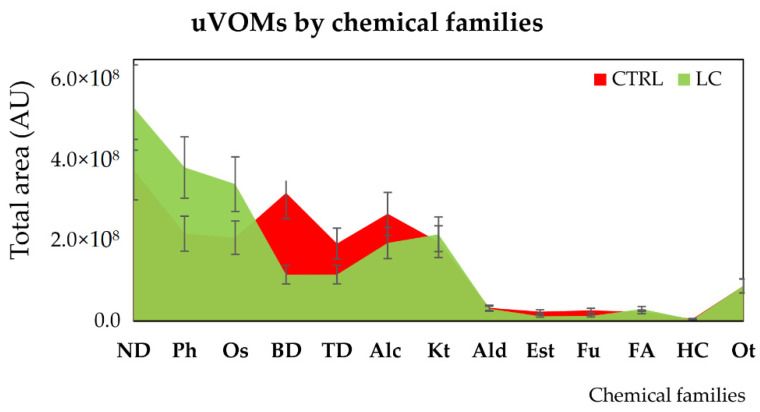
Distribution of the uVOMs identified in control (CTRL) and lung cancer (LC) individuals by chemical families. Legend: Alc—alcohols; Ald—aldehydes; AU—arbitrary units; BD—benzene derivatives; Est—esters; FA—fatty acids; Fu—furans; HC—hydrocarbons; Kt—ketones; ND—naphthalene derivatives; Ot—others; Os—organosulfurs; Ph—phenols; TD—terpenic derivatives.

A large variety of chemical structures and classes—namely, aldehydes, acids, ketones, sulfur compounds, furanic compounds, terpenic compounds, benzene derivates, phenols, among others—were identified in the volatomic data obtained. Overall, there are appreciable differences in the abundance of certain classes between the control and LC patients—including naphthalene derivatives, phenols, and organosulphurs augmented in the LC group. In contrast, benzene and terpenic derivatives were found to be more prevalent in the CTRL group, while there are not statistical differences among alcohols, ketones, aldehydes, esters, furans, and hydrocarbons (Figure 3).

Overall, 147 VOMs were identified in the urine samples (uVOMs, detailed data available in the Table S1). Many of these uVOMs have been previously reported in different reports involving urine samples of healthy and cancer patients [28,29,36,37,38,39,40]. Taking into consideration that the recruited subjects belong to a diverse genetic pool in the Indian subcontinent, such a result is evidence of the robustness of the methodology that we previously optimised [37,38,39,40]. The interplay of genetics, diet, and environment certainly interferes with the urinary volatomic profiles from subjects between such different human populations as we previously show for saliva samples from healthy and breast cancer patients in Madeira Island (Portugal, South Europe) and Pune (Indian subcontinent) [41]. 

### 2.2. Volatomic Data Processing Using Advanced Statistical Analysis

**Figure 4:**
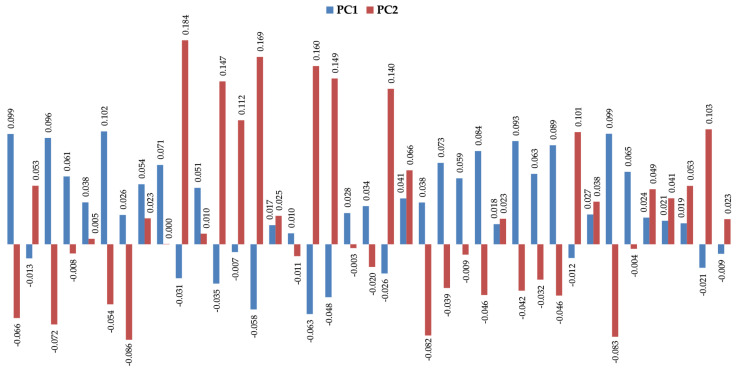
Profile of the two first principal components (PC1 vs. PC2) for the most significative uVOMs identified in this work.

The volatomic data obtained (Table S1) suggest important variations between the target groups that can be easily observed in the principal components analysis (PCA) shown in Figure 4.

**Figure 5:**
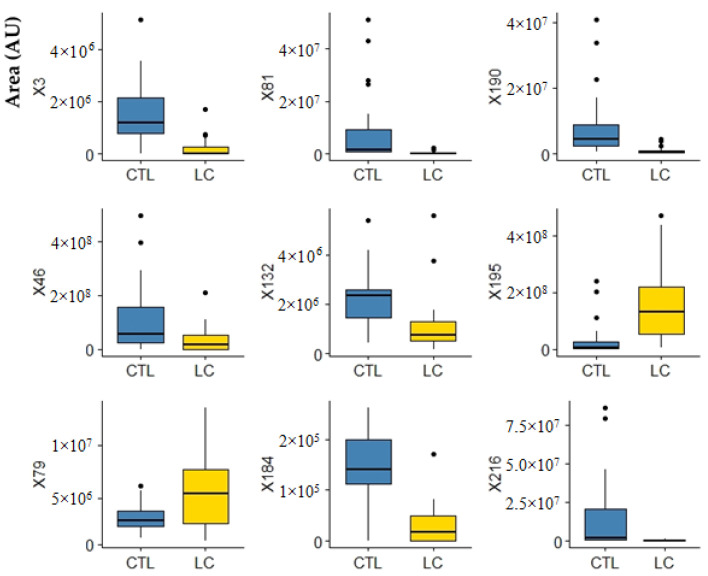
Boxplots of the most important variables (uVOMS) for the discrimination of LC patients from control subjects (CTRL). Legend: AU—arbitrary units; CTRL—control group; LC—lung cancer; X3—furan; X46—o-cymene; X79—furfural; X81—linalool oxide; X132—viridiflorene; X184—2-bromophenol; X190—tricyclazole; X195—p-cresol; X216—1-(4-hydroxy-3,5-di-tert-butylphenyl)-2-methyl-3-morpholinopropan-1-one.

These results led us to further process the data obtained using multivariate statistical analysis (R software [42]) to identify significant metabolites able to discriminate the LC patients from the healthy individuals. Support-vector machine (SVM), random forest (RF), multilayer perceptron (MLP), and partial least squares discriminant analysis (PLS-DA) were used as classification algorithms to separate the groups under study. The performance of the classification models was evaluated and obtained from the 10-fold cross validation methodology, repeated 10 times and from the accuracy, sensitivity, specificity, area under the curve (AUC), and the ROC curve. The variable selectors used were correlation-based feature selection (CFS), which selects a subset of variables; and the F-score selector, which assigns an importance value to the variables, generating an importance ranking. As a result, despite all classifiers retrieving good results, the best was obtained with MLP (accuracy, sensitivity, and specificity data are mentioned in Table S2 and respective ROC curves in Figure S1). Following this, the CFS variable selector was applied to the volatomic data and 17 uVOMs were selected as the most important ones (Table 2), being able to deliver higher discriminant accuracy and AUC for the target groups (Table S2 and Figure S1). F-score was also used to select the most important variables, retrieving 25 uVOMs of different importance (F-scores values, Figure S2). The 25 variables from the F-score selector (Table 2) were used to obtain the equivalent number of subsets of variables containing only the most important variable, then the two most important, then the three most important, until all 25 variables form the last group. These 25 subsets of uVOMs were then processed using SVM, RF, MLP, and PLS-DA and the result obtained (Figure S2) shows that SVM and RF produce the best classifications, retrieving 20 and 15 uVOMS that were able to discriminate LC patients from control subjects with 96.67% accuracy. Finally, the uVOMS that were simultaneously reported as the most important using the different CFS and F-score upon SVM were observed to allow the discrimination of the target groups with 100% accuracy using SVM and MLP (Table 2 and Table S2). This set of nine uVOMS includes furan, o-cymene, furfural, linalool oxide, viridiflorene, 2-bromo-phenol, tricyclazole, 4-methyl-phenol, and 1-(4-hydroxy-3,5-di-tert-butylphenyl)-2-methyl-3-morpholinopropan-1-one, and their respective boxplots are presented in Figure 5.

Part of these uVOMs are related to the diet and thus their interpretation as putative biomarkers for lung cancer is not easy to attain. O-cymene (X46, Figure 5), for instance, is typically found in citrus fruits and hardly found elsewhere [43]. For this reason, *O*-cymene has been proposed as a putative biomarker of citrus ingestion and effectively it has been detected in the urine samples from all recruited CTRL (28) and in 20 out of 27 LC samples. Moreover, it has been previously reported in saliva and faeces [43] and recently was indicated to contribute for the discrimination of alcoholic cirrhotic patients from healthy volunteers [44]. Similarly, linalool oxide (X81, Figure 5) is a monoterpenoid compound commonly found in many aromatic plants and has been previously reported in the urine of different cancer patients and respective control subjects [37,38]. Viridiflorene (X132, Figure 5) has also a dietary origin. This compound is an aromadendrane sesquiterpenoid present in many aromatic plants and spices widely used in Indian cuisine—such as sweet basil, sweet marjoram, oregano, and rosemary [43]—and it has been previously reported in saliva [43,45]. In turn, furan (X3, Figure 5), results most probably from thermal degradation of natural food components and it is widely present in processed commercial foods [43]. P-cresol (X195, Figure 5) is produced by intestinal microflora in humans during the aromatic amino acid metabolism [43] and their levels in urine strongly correlate with the levels of proteins in the diet. Nevertheless, human metabolism is very complex and cancer development certainly makes this scenario even more difficult to understand. Therefore, it is plausible that certain metabolites may result from different biochemical pathways in the human body as well as their levels change due to the cancer development and progression. Regarding this, we have previously found that furan, linalool oxide and p-cresol (X3, X81, and X195 in Figure 5, respectively) were more abundant in the urine of BC patients [38,39]. In fact, growing evidence points to furan as a possible human carcinogen [46]. Despite its short half-life, furan was shown to accumulate in the livers of rats and mice where is metabolised to the reactive cis-but-2-ene-1,4-dialdehyde that binds covalently to DNA, triggering hepatocellular adenomas/carcinomas development [47]. It is therefore very relevant to point out that furan and derivatives have been reported in many studies involving the exhaled breath composition of LC patients [48]. Another uVOM identified in this work, 2-bromophenol (X184, Figure 5), has been previously reported as a putative urinary volatile biomarker for BC [29]. This is a metabolite of polybrominated diphenyl ethers (PBDEs) that became widespread in the environment due to the massive use of wood preservatives and fire retardants [49], but it is also a primary metabolite essential for cell growth [43]. Tricyclazole (X190 in Figure 5) is most probably an environmental contaminant. This is a fungicide used against *Pyricularia oryzae*, the heterothallic ascomycetous pathogen responsible for the rice blast, the most destructive disease in rice crops [50]. High concentrations of tricyclazole have been reported in drinking water treatment plants in the Yangtze River Delta [51], which is one of the major rice-producing areas in China [52]. Similarly, the recruited subjects in this study live nearby Pune, India, where there is also rice production, which may explain why this compound was detected in the urine of the recruited subjects. In a previous study involving BC patients and controls in Indian cohorts, we found that 1-(4-hydroxy-3,5-di-tert-butylphenyl)-2-methyl-3-morpholinopropan-1-one (X216 in Figure 5) was more abundant in the urine of control subjects, being one of the 14 uVOMs statistically relevant for the discrimination between both groups [39]. Unfortunately, the information currently available in the literature about this metabolite is scarce.

### 2.3. Metabolic Pathways

**Figure 6:**
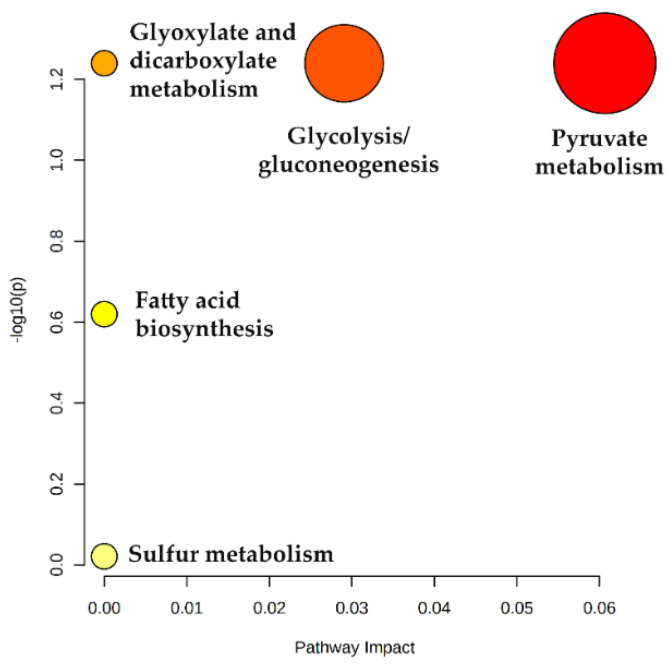
Metabolic pathway analysis showing dysregulated metabolic pathways in LC patients. Pathway impact reflects the importance (cumulative percentage of the matched metabolite nodes) that the statistically significant uVOMs identified in this work (as assessed by log *p* values) have in the different metabolic pathways.

To get insights into the altered metabolic pathways in the LC patients recruited in this study, a metabolic pathway analysis was performed using the MetPA tool in Metaboanalyst 5.0 Pathway topology [53]. The list of uVOMs identified as statistically significant and differentially regulated was uploaded and the MetPA tool identified the enriched biochemical pathways that were differentially affected in the target groups. The result obtained is expressed as a bubble plot of log(*p*) versus pathways impact (Figure 6, detailed data available in Table S3) and show that pyruvate metabolism and glycolysis/gluconeogenesis are the pathways most affected, being excessively active in LC in comparison to the healthy controls.

**Figure 7:**
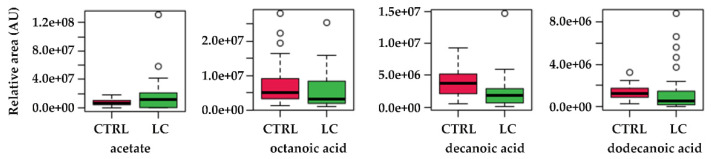
Boxplots of the variations found for the most relevant metabolites identified in the pathway topology analysis. CTRL—control subjects, LC—lung cancer patients.

Not surprisingly, acetate (acetic acid) was identified as a major player in this metabolic shift in LC. In fact, different studies show that tumour cells can use acetate both as bioenergetic fuel, and as a nutritional source to support lipid biosynthesis [54]. As reviewed by Bose et al. [55], while during normal metabolism, cells use acetyl-CoA derived from glucose, under the hypoxic conditions verified in tumour microenvironments, cancer cells activate a de novo pathway for acetate production from pyruvate, the end product of glycolysis. This glucose-independent acetate metabolism has been reported to promote melanoma cell survival and tumour growth [56]. In this process, the nucleocytosolic acetyl-CoA synthetase enzyme, ACSS2, has been described as the supplier of acetyl-CoA for tumours by capturing acetate as a carbon source [57]. Previously, we found augmented levels of acetic acid in the urine samples of breast cancer patients [39] and Filipiak et al. [58] reported altered levels of acetic acid in the lung cancer tissue, although not at statistically significant levels. Similarly, our data also points to augmented levels of acetate in the urine of LC patients (Figure 7). Dodecanoic, decanoic, and octanoic acids were also identified in the metabolic pathways as main contributors for the fatty acid biosynthesis deregulation, although their individual variation is not so evident as acetate (Figure 7). Fatty acids have a key role as structural components of the membrane matrix but can also act as secondary messengers and serve as fuel sources for energy production, and these features are also very relevant under cancer development [59]. Regarding this, very recently Qi, Wu, Chen, Zhang, Zhou, Mao, Li, Li, Chen, Huang, and Huang [18] reported that plasma levels of saturated fatty acids (SFAs), such as dodecanoic acid, were significantly decreased in cancer groups. In turn, altered plasma levels of decanoic acid were reported as a putative new diagnostic biomarker in colorectal cancer [60] and the serum level of octanoic acid were found to predict the efficacy of chemotherapy against the same type of cancer [61].

## 3. Materials and Methods

### 3.1. Reagents and Materials

Sodium chloride (NaCl) and hydrogen chloride (HCl) were purchased from Merck (Darmstadt, Germany). Solid phase micro-extraction manual holder fibre CAR/PDMS (75 µm) was purchased from Supelco (Bellefonte, PA, USA).

### 3.2. Subjects and Sample Collection

LC urine samples were collected from subjects (*n* = 28, age = 55.5 ± 12.4 years, range 31–73 years, 16 male and 12 female, 11 smokers) with a recent LC diagnosis made by the Malignant Disease Treatment Centre (MDTC), Unit of the Military Hospital-Cardio Thoracic Centre (MH-CTC), Armed Forces Medical College (AFMC), Pune, India. Additionally, selected LC patients were devoid from any other comorbidity like hypertension, asthma, or diabetes. Stratification of the different LC subtypes include 12 metastatic adenocarcinoma, 6 non-small cell lung carcinoma, 5 metastatic carcinoma, 3 squamous cell carcinoma, and 2 alveolar carcinoma. In turn, the volunteered healthy subjects (CTRL) (*n* = 27, age = 36.1 ± 9.1 years, range 24–56 years, 18 male and 9 female, 9 smokers) were eligible to participate in the study if they were 18 years older and had no previously diagnosed cancer or any other comorbidities. Samples from these healthy controls were obtained through the health check-up camp organized by the MDTC, MH-CTC, AFMC, Pune. Smoking habits were thoroughly checked to distinguish between non-smoker and ex-smoker subjects. This study was approved by the institutional ethics committee of the AFMC and the National Centre for Cell Science (NCCS). All the participants in this study were informed about the investigation and informed consent approval was obtained from the patients before sample collection following the Declaration of Helsinki guidelines (DoH, 2008).

The characteristics of all subjects are summarized in Table 1. Each subject (LC patient or healthy volunteer) provided a sample of first-morning urine (after overnight fasting) in a 50 mL sterile glass container. The samples were aliquoted (4 mL) in 8 mL vials and frozen at −80 °C until needed for experiments. Before the extraction procedure, the pH value of urine used for each extraction was adjusted to 3. 

### 3.3. Analytical Procedure and Sample Preparation

Urine samples aliquots were thawed and added 0.5 mL of hydrochloric acid (5M) and 0.8 g NaCl (20% NaCl *w*/*v*). Under magnetic agitation, at 50 ± 1 °C, SPME fibre coating was exposed in the sample HS for 60 min. Finally, the SPME fibre was manually inserted into the GC injector at 250 °C, in splitless mode, for 6 min, to desorb the extracted uVOMs. All experiments were performed in triplicate, including blanks assays corresponding to the analysis of coating fibres not submitted to any extraction procedure.

### 3.4. Gas Chromatography-Quadrupole Mass Spectrometry Analysis (GC-qMS)

GC-qMS analysis was performed was previously reported [39,40]. Briefly, extracted uVOMS were chromatographically separated and identified using an Agilent 7890B gas chromatograph (Palo Alto, CA, USA) coupled to an Agilent 5977A quadrupole inert mass selective detector and a BP-20 (SGE, Darmstadt, Germany) fused silica capillary column (60 m × 0.25 mm × 0.25 μm) installed in the GC oven. The chromatographic temperature gradient, in a total run time of 87 min, was the following: 5 min at 45 °C, gradually ramped up to 150 °C at 2 °C min^−1^, 10 min hold time and new ramp to 220 °C (15 °C min) and held for 15 min. The mobile phase/carrier gas used was ultra-high purity helium gas (99.999%, Prama Instruments, Mumbai, India), with a flow rate of 1 mL min^−1^. All the samples were acquired in duplicates. The operating temperatures of the transfer line, quadrupole, and electron impact ionisation source were 250, 150, and 230 °C, respectively. Data acquisition was performed in full scan mode in the mass range of 30 to 300 m/z and 70 eV was applied for the electron impact to record the mass spectra. The identification of the metabolites was performed using the Agilent ChemStation data analysis software (Palo Alto, CA, USA) coupled with the NIST11 mass spectral library. A match score over 80% was used with the metabolite identification hits from the library search and further chromatogram integration to generate peak areas was performed using ChemStation data analysis software (version F.01.00.1903). To obtain the reference retention indices for the identified uVOMs and allow their comparison with the Kovats indices available in the literature for similar experimental conditions, the C8–C20 n-alkanes series were analysed under the same experimental conditions. To improve data reliability, VOCs showing missing values >80% across all the samples were not considered for further analysis.

### 3.5. Statistical Analysis

Multivariate statistical analysis was performed using R software (version 4.0.5) [42]. Support vectors machine (SVM), random forests (RF), multilayer perceptron (MLP), and partial least squares discriminant analysis (PLS-DA) were used as classification algorithms. The performance of the classification models was obtained from the 10-fold cross validation methodology, repeated 10 times and from the accuracy, sensitivity, specificity, area under the curve (AUC), and the ROC curve. Two variable selectors were used: correlation-based feature selection (CFS), which selects a subset of variables; and the F-score selector, which assigns an importance value to the variables, generating an importance ranking. The metabolic pathway analysis was performed using the MetPA tool in Metaboanalyst 3.0. Pathway topology [62]. This tool combines metabolite set enrichment analysis (MSEA) and pathway topology analysis to identify biochemical pathways that are differentially affected in the target groups. This is done using around 6300 metabolite sets that compose the *Homo sapiens* libraries. Following this analysis, a list of uVOMs identified as statistically significant and differentially regulated, is uploaded in the enrichment analysis module to identify the enriched biochemical pathways.

## 4. Conclusions

A total of 147 uVOMs of several chemistries were identified in the intervention groups, including naphthalene derivatives, phenols, and organosulphurs augmented in the LC group. In contrast, benzene and terpenic derivatives were found more prevalent in the control group (CTRL). The volatomic data obtained were processed using advanced statistical analysis, namely support vector machine (SVM), random forest (RF), multilayer perceptron (MLP), and partial least square discriminative analysis (PLS-DA) methods. Nine from 147 uVOMs namely, furan, o-cymene1-methyl-2-(1-methylethyl)-benzene, furfural, linalool oxide, viridiflorene, 2-bromo-phenol, tricyclazole, 4-methyl-phenol, and 1-(4-hydroxy-3,5-di-tert-butylphenyl)-2-methyl-3-morpholinopropan-1-one were identified with a high potential to discriminate LC patients from control subjects, most of them are related with the dietary habits of the subjects. The metabolic pathway analysis of the data obtained identified several altered biochemical pathways in LC mainly affecting glycolysis/gluconeogenesis, pyruvate metabolism, and fatty acid biosynthesis. Moreover, acetate and octanoic, decanoic, and dodecanoic fatty acids were identified as the key metabolites responsible for such deregulation.

Tricyclazole, a fungicide used against rice fungal infections, was found in all samples analysed, suggesting that this pesticide is already widely contaminating the different habitats in which the subjects were recruited. Whilst part of the uVOMs identified in this work are related to the dietary habits of the recruited subjects, their metabolism is certainly affected by cancer development and progression and additional experiments with a higher number of subjects in different stages of the disease will be very important to assess their potential as putative LC biomarkers. Overall, the use of the volatomic methodology to study the uVOMs across various biofluids holds a promising potential for population-wide screening programs across a variety of diseases, especially for low-income countries. This strategy could be highly beneficial to people when more in-depth and controlled study designs in this area of research are implemented in the coming future.

## Figures and Tables

**Table 1:** Characterisation of the subjects recruited in terms of number, age, gender, and smoking habits.

Subject Variables	Control Subjects (CTRL)	LC Patients
Number	27	28 *
Mean age (range)	36.1 (25–52)	55.5 (27–73)
Gender	16 male, 11 female	18 male, 9 female
Smokers	9	12

Legend: LC—lung cancer patients, * LC subtypes and their counts: metastatic adenocarcinoma of lung = 12; non-small cell lung carcinoma = 6; metastatic lung carcinoma = 5; squamous cell carcinoma of lung = 3, alveolar carcinoma = 2.

**Table 2:** Most important variables identified using the different classification algorithms.

Label ^a^	Volatiles	CFS	F-Score	F-Score	F-Score
				SVM	RF
3 ^b^	Furan	X	X	X	X
46	o-Cymene	X	X	X	X
64	p-Cymenene		X	X	
78	Acetic acid	X			
79	Furfural		X	X	X
81	Linalool oxide	X	X	X	X
83	2,6-Dimethyl-7-octen-2-ol	X			
132	Viridiflorene	X	X	X	X
133	β-Guaiene	X			
149	3,6-dimethyl-1H-indazole		X		
152	1-(3,5-Bis-trifluoromethylphenyl)ethanol	X			
153	Benzoyl isocyanate		X	X	
158	1,2,3,3-Tetramethyl-cyclopenten-4-one		X	X	
162	methoxy-phenyl-oxime	X			
164	4-(1-Methylethyl)-benzaldehyde		X		
165	2,4,6-Trimethylbenzyl alcohol		X	X	
177	2-Methyl-1-(1,1-dimethylethyl)-2-methyl-1,3-propanediyl ester propanoic acid		X	X	
179	α-Calacorene		X	X	
184	2-Bromo-phenol	X	X	X	X
187	4-(2,6,6-trimethylcyclohexa-1,3-dienyl)but-3-en-2-one		X	X	
188	Phenol				
190	Tricyclazole	X	X	X	X
191	3,8-Dimethyl-5-(1-methylethyl)-1,2-naphthalenedione		X		
195	p-Cresol	X	X	X	X
198	4,4,5,8-Tetramethyl-4H-1-benzopyran		X		
200	Indanone		X	X	
201	Nonanoic acid		X		
203	2-[(2-ethoxy-3,4-dimethyl-2-cyclohexen-1-ylidene)methyl]-furan	X			
207	2-Bromo-4-(1,1-dimethylethyl)-phenol		X	X	
208	muurolane		X	X	
212	2,3-Dihydro-3,3,4,5-pentamethyl-1H-inden-1-one		X	X	
216	1-(4-Hydroxy-3,5-di-tert-butylphenyl)-2-methyl-3-morpholinopropan-1-one	X	X	X	X
219	Dodecanoic acid	X			

^a^ Number of identified uVOM, listed in Table S1 (Supplementary Material); ^b^ uVOMs indicated in bold were simultaneously reported as the most important using the different CFS and F-score upon SVM, allowing the discrimination of the target groups with 100% accuracy using SVM and MLP.

## Data Availability

The data presented in this study are available on request from the corresponding author on mentioning genuine requirements, because of it’s usage in the ongoing study.

## Supplementary Materials

The following are available online at https://www.mdpi.com/article/10.3390/metabo12010036/s1, Figure S1: Performance of the variables identified with the different algorithms described in Table 2 and Table S3; Figure S2: Results of the classification of the 25 groups generated from the F-score classification of the uVOMs identified in this work; Table S1: GC-qMS peak areas of the volatile metabolites identified in LC patients and healthy subjects organised by chemical family; Table S2: Results of the target groups discrimination following the use of different classification algorithms; Table S3: Pathway analysis results.
