# Supplementary material for: Urinary Volatomic Expression Pattern: Paving the Way for Identification of Potential Candidate Biosignatures for Lung Cancer
Source: Metabolites. 2022 Jan 4;12(1):36. doi: 10.3390/metabo12010036 (PMC8780352; doi:10.3390/metabo12010036)
Supplement: Supplementary file 1 [file metabolites-12-00036-s001.zip › metabolites-1473663-supplementary.pdf]

**Table S1.** GC-qMS peak areas of the volatile metabolites identified in LC patients and healthy subjects (CTRL) organized by chemical family

| label | Chemical Classes / Metabolites | CTRL | | | | LC | | | | Fold change | |
|---|---|---|---|---|---|---|---|---|---|---|---|
| | | AVG | Max | Min | Freq (%) | AVG | Max | Min | Freq (%) | | |
| | **Alcohols** | | | | | | | | | LC-CTRL | % variation |
| 40 | Isopentanol | 1.7E+05 | 6.0E+05 | 2.7E+04 | 100 | 2.9E+05 | 3.2E+06 | 0.0E+00 | 89 | 1.2E+05 | 42.1 |
| 62 | 1-Hexanol | 1.0E+05 | 2.5E+05 | 0.0E+00 | 93 | 1.2E+05 | 9.1E+05 | 0.0E+00 | 82 | 1.8E+04 | 15.2 |
| 76 | 3,7-Dimethyl-3-octanol | 2.3E+05 | 5.1E+05 | 1.3E+05 | 100 | 1.8E+05 | 6.2E+05 | 1.8E+04 | 100 | -5.5E+04 | -31.0 |
| 77 | 2-Ethyl-1-hexanol | 3.0E+05 | 5.4E+05 | 1.1E+05 | 100 | 2.2E+05 | 5.9E+05 | 0.0E+00 | 93 | -8.2E+04 | -36.8 |
| 83 | 6,10-Dihydromyrcenol | 1.2E+07 | 1.1E+08 | 2.6E+05 | 100 | 5.4E+05 | 2.4E+06 | 0.0E+00 | 96 | -1.2E+07 | -2155.9 |
| 101 | 3,7-Dimethyl-1,6-octadien-3-ol | 1.1E+05 | 7.0E+05 | 0.0E+00 | 93 | 4.1E+04 | 2.5E+05 | 0.0E+00 | 64 | -6.6E+04 | -160.6 |
| 136 | 2-Furanmethanol | 2.0E+05 | 6.9E+05 | 2.4E+04 | 100 | 1.6E+05 | 9.7E+05 | 0.0E+00 | 86 | -4.9E+04 | -31.7 |
| 152 | 1-(3,5-Bis-trifluoro-methylphenyl)ethanol | 7.5E+04 | 5.8E+05 | 0.0E+00 | 44 | 1.7E+07 | 3.4E+08 | 0.0E+00 | 79 | 1.7E+07 | 99.6 |
| 165 | 2,4,6-Trimethylbenzyl alcohol | 2.4E+06 | 1.3E+07 | 6.7E+03 | 100 | 2.8E+05 | 2.1E+06 | 0.0E+00 | 86 | -2.2E+06 | -764.9 |
| 189 | 1,2-Decanediol | 2.5E+08 | 4.0E+08 | 4.5E+07 | 100 | 1.7E+08 | 4.4E+08 | 8.8E+06 | 100 | -7.6E+07 | -43.2 |
| | **TOTAL AREA** | **2.7E+08** | | | | **1.9E+08** | | | | | |
| | **Aldehydes** | | | | | | | | | | |
| 41 | 2-Hexenal | 6.6E+05 | 4.4E+06 | 7.2E+04 | 100 | 4.9E+05 | 1.8E+06 | 9.5E+03 | 100 | -1.7E+05 | -35 |

| | | | | | | | | | | | |
|---|---|---|---|---|---|---|---|---|---|---|---|
| 79 | Furfural | 3.1E+06 | 6.1E+06 | 1.2E+06 | 100 | 5.5E+06 | 1.4E+07 | 9.3E+05 | 100 | 2.3E+06 | 42.6 |
| 87 | Decanal | 2.3E+07 | 9.3E+07 | 1.5E+06 | 100 | 2.3E+07 | 5.4E+07 | 2.4E+05 | 100 | -6.2E+05 | -2.7 |
| 164 | Cuminaldehyde | 5.7E+06 | 3.3E+07 | 1.1E+06 | 100 | 1.9E+06 | 7.1E+06 | 2.5E+05 | 100 | -3.8E+06 | -205.2 |
| | **TOTAL AREA** | **3.3E+07** | | | | **3.1E+08** | | | | | |
| | **Benzene Derivatives** | | | | | | | | | | |
| 46 | *o*-Cymene | 1.2E+08 | 5.0E+08 | 3.0E+04 | 100 | 3.6E+07 | 2.1E+08 | 0.0E+00 | 75 | -8.0E+07 | -225.1 |
| 52 | 1-Isopropyl-3-methylbenzene | 1.4E+07 | 1.6E+08 | 0.0E+00 | 85 | 3.8E+06 | 9.0E+07 | 0.0E+00 | 86 | -1.0E+07 | -268.5 |
| 57 | Hemimellitene | 5.3E+06 | 1.7E+07 | 0.0E+00 | 96 | 3.3E+06 | 2.0E+07 | 0.0E+00 | 93 | -2.0E+06 | -60.2 |
| 64 | p-Cymenene | 5.1E+07 | 1.6E+08 | 3.8E+04 | 100 | 1.1E+07 | 3.4E+07 | 0.0E+00 | 93 | -4.0E+07 | -368.6 |
| 68 | Toluene | 5.5E+05 | 7.5E+06 | 1.3E+03 | 100 | 2.1E+05 | 1.7E+06 | 0.0E+00 | 89 | -3.4E+05 | -161.4 |
| 71 | (1,4-Dimethylpent-2-enyl)benzene | 2.0E+06 | 9.5E+06 | 0.0E+00 | 74 | 1.4E+06 | 1.2E+07 | 0.0E+00 | 93 | -6.7E+05 | -49.3 |
| 72 | Benzene, 1-butenyl-, (Isomer)- | 3.2E+07 | 1.8E+08 | 0.0E+00 | 93 | 1.4E+07 | 6.3E+07 | 0.0E+00 | 86 | -1.7E+07 | -121 |
| 84 | 1,2,4,5-Tetramethylbenzene | 8.4E+06 | 2.6E+07 | 3.5E+05 | 100 | 5.0E+06 | 1.4E+07 | 0.0E+00 | 96 | -34E+06 | -68.4 |
| 98 | 1H-Trindene, 2,3,4,5,6,7,8,9-octahydro-1,1,4,4,9,9-hexamethyl- | 1.6E+06 | 6.3E+06 | 2.5E+05 | 100 | 2.9E+06 | 1.3E+07 | 1.3E+05 | 100 | 1.3E+06 | 43.8 |
| 153 | Benzoyl isocyanate | 1.4E+06 | 4.2E+06 | 1.3E+05 | 100 | 6.4E+05 | 3.2E+06 | 1.3E+05 | 100 | -72E+05 | -112.9 |
| 162 | Oxime-,methoxy-phenyl- | 1.9E+06 | 8.8E+06 | 4.5E+05 | 100 | 1.1E+06 | 7.1E+06 | 1.9E+03 | 100 | -7.4E+05 | -65.8 |
| 176 | α-Bromomesitylene | 9.5E+06 | 7.5E+07 | 1.9E+05 | 100 | 6.2E+06 | 4.4E+07 | 0.0E+00 | 93 | -3.3E+06 | -53.9 |

| | | | | | | | | | | | |
|---|---|---|---|---|---|---|---|---|---|---|---|
| 190 | Tricyclazole | 8.3E+06 | 4.1E+07 | 7.4E+05 | 100 | 8.5E+05 | 4.5E+06 | 5.4E+04 | 100 | -7.5E+06 | -871.7 |
| 198 | 4,4,5,8-tetramethylchromene | 4.3E+07 | 1.9E+08 | 8.2E+06 | 100 | 1.8E+07 | 1.2E+08 | 7.8E+05 | 100 | -2.5E+07 | -135.9 |
| 202 | Eugenol | 1.7E+07 | 1.2E+08 | 1.0E+05 | 100 | 1.6E+06 | 1.2E+07 | 3.6E+04 | 100 | -1.5E+07 | -941.6 |
| 212 | 1H-Inden-1-one, 2,3-dihydro-3,3,4,5-pentamethyl- | 1.4E+05 | 3.2E+05 | 3.5E+04 | 100 | 7.2E+04 | 2.9E+05 | 0.0E+00 | 82 | -6.8E+04 | -94.2 |
| 218 | Indole | 2.7E+06 | 2.5E+07 | 8.9E+04 | 100 | 2.0E+05 | 5.6E+05 | 0.0E+00 | 82 | 4.2E+06 | 46.6 |
| | **TOTAL AREA** | **3.2E+08** | | | | **1.2E+08** | | | | | |
| | **Esters** | | | | | | | | | | |
| 125 | Methyl benzoate | 1.3E+06 | 8.0E+06 | 7.3E+04 | 100 | 1.2E+06 | 5.4E+06 | 1.7E+05 | 100 | -1.2E+05 | -10.4 |
| 177 | Propanoic acid, 2-methyl-, 1-(1,1-dimethylethyl)-2-methyl-1,3-propanediyl ester | 2.2E+07 | 5.4E+07 | 2.7E+06 | 100 | 8.9E+06 | 2.3E+07 | 1.6E+05 | 100 | -1.3E+07 | -142.2 |
| 220 | Diisobutyl phthalate | 2.7E+05 | 1.5E+06 | 0.0E+00 | 48 | 2.0E+06 | 1.5E+07 | 0.0E+00 | 86 | 1.7E+06 | 86.4 |
| | **TOTAL AREA** | **2.3E+07** | | | | **1.2E+07** | | | | | |
| | **Furans** | | | | | | | | | | |
| 3 | Furan | 1.6E+06 | 5.1E+06 | 0.0E+00 | 96 | 2.4E+05 | 1.7E+06 | 0.0E+00 | 46 | -1.3E+06 | -551.4 |
| 13 | 2,5-Dimethylfuran | 7.2E+05 | 2.3E+06 | 0.0E+00 | 89 | 7.0E+05 | 2.3E+06 | 3.3E+04 | 100 | -1.6E+04 | -2.3 |
| 15 | 2-Ethyl-5-methylfuran | 4.5E+06 | 3.7E+07 | 3.0E+05 | 100 | 1.9E+06 | 1.2E+07 | 8.3E+04 | 100 | -2.6E+06 | -131.8 |
| 44 | 2,2-Dimethyl-5-(1 -methyl-1-propenyl)-tetrahydrofuran | 2.0E+06 | 8.2E+06 | 1.5E+05 | 100 | 8.0E+05 | 3.6E+06 | 0.0E+00 | 96 | -1.2E+06 | -149.2 |

| | | | | | | | | | | | |
|---|---|---|---|---|---|---|---|---|---|---|---|
| 61 | 2-Methyl-3-(methylthio)furan | 6.4E+05 | 1.5E+06 | 3.6E+04 | 100 | 8.0E+05 | 3.2E+06 | 0.0E+00 | 93 | 1.6E+05 | 20.2 |
| 75 | 5-Ethenyltetrahydro-5-trimethyl-trans-2-furanmethanol | 9.0E+05 | 3.9E+06 | 7.1E+04 | 100 | 8.6E+05 | 6.0E+06 | 1.6E+04 | 100 | -3.2E+04 | -3.8 |
| 81 | Linalool oxide | 8.2E+06 | 5.1E+07 | 2.6E+05 | 100 | 4.2E+05 | 2.3E+06 | 0.0E+00 | 96 | -7.8E+06 | -1853.2 |
| 105 | 5-Methyl-2-furancarboxaldehyde | 3.7E+06 | 9.7E+06 | 9.9E+05 | 100 | 5.1E+06 | 3.6E+07 | 6.3E+05 | 100 | 1.4E+06 | 28.3 |
| 122 | Dihydro-5-methyl-2(3H)-furanone | 3.4E+05 | 1.4E+06 | 4.8E+03 | 100 | 2.7E+05 | 1.5E+06 | 0.0E+00 | 79 | -7.3E+04 | -27.4 |
| 145 | Benzofuran, 4,7-dimethyl- | 2.3E+06 | 8.0E+06 | 3.0E+05 | 100 | 1.5E+06 | 7.9E+06 | 8.0E+04 | 100 | -8.4E+05 | -56.7 |
| 203 | 2-[(*E*)-(2-ethoxy-3,4-dimethylcyclo-hex-2-en-1-ylidene)methyl]furan | 1.6E+06 | 1.2E+07 | 4.5E+04 | 100 | 9.8E+04 | 6.3E+05 | 1.1E+04 | 100 | -1.5E+06 | -1538.7 |
| | **TOTAL AREA** | **2.6E+07** | | | | **1.3E+07** | | | | | |
| | **Hydrocarbons** | | | | | | | | | | |
| 104 | 2-Methyl-1-butene | 1.7E+05 | 5.9E+05 | 0.0E+00 | 81 | 1.1E+05 | 3.6E+05 | 0.0E+00 | 82 | -5.8E+04 | -53.6 |
| 159 | 4,5-Dehydro-Isolongifolene | 2.2E+06 | 1.4E+07 | 2.3E+05 | 100 | 1.4E+06 | 6.0E+06 | 3.9E+04 | 100 | -8.1E+05 | -57.6 |
| 183 | 4,5,9,10-Dehydroisolongifolene | 2.9E+06 | 3.2E+07 | 7.9E+04 | 100 | 2.0E+06 | 1.2E+07 | 0.0E+00 | 89 | -8.8E+05 | -43.0 |
| | **TOTAL AREA** | **5.3E+06** | | | | **3.6E+06** | | | | | |
| | **Ketones** | | | | | | | | | | |
| 4 | Acetone | 1.2E+07 | 2.3E+07 | 1.5E+06 | 100 | 1.8E+07 | 9.5E+07 | 1.2E+06 | 100 | 6.2E+06 | 34.8 |
| 8 | Butanone | 4.1E+06 | 1.4E+07 | 5.4E+05 | 100 | 7.7E+06 | 3.6E+07 | 2.7E+05 | 100 | 3.6E+06 | 46.8 |
| 14 | 2-Pentanone | 2.1E+07 | 9.4E+07 | 1.7E+06 | 100 | 5.0E+07 | 2.4E+08 | 1.2E+06 | 100 | 2.9E+07 | 58.2 |

| | | | | | | | | | | | |
|---|---|---|---|---|---|---|---|---|---|---|---|
| 22 | 3-Hexanone | 2.1E+06 | 6.0E+06 | 1.3E+05 | 100 | 3.6E+06 | 1.4E+07 | 2.0E+05 | 100 | 1.5E+06 | 40.9 |
| 27 | 4-Heptanone | 6.1E+07 | 1.8E+08 | 5.6E+06 | 100 | 7.0E+07 | 3.2E+08 | 0.0E+00 | 96 | 9.0E+06 | 12.8 |
| 32 | 2-Heptanone | 2.6E+06 | 5.8E+06 | 1.1E+05 | 100 | 6.3E+06 | 7.4E+07 | 1.8E+05 | 100 | 3.7E+06 | 58.8 |
| 60 | 3-Ethylcyclopentanone | 2.0E+05 | 1.2E+06 | 0.0E+00 | 93 | 3.3E+05 | 4.5E+06 | 0.0E+00 | 89 | 1.3E+05 | 38.9 |
| 69 | 3,5,5-Trimethyl-2-cyclohexen-1-one | 7.2E+05 | 2.1E+06 | 1.3E+05 | 100 | 1.4E+06 | 5.4E+06 | 3.3E+04 | 100 | 6.6E+05 | 48.0 |
| 82 | 6-Camphenone | 2.8E+05 | 1.1E+06 | 3.2E+04 | 100 | 2.2E+05 | 1.1E+06 | 0.0E+00 | 96 | -6.4E+04 | -29.4 |
| 86 | 2-Decanone | 2.3E+07 | 9.3E+07 | 1.4E+06 | 100 | 1.9E+07 | 5.4E+07 | 0.0E+00 | 93 | -4.8E+06 | -25.7 |
| 91 | 2-Acetylfuran | 9.6E+06 | 3.9E+07 | 1.3E+06 | 100 | 1.1E+07 | 3.0E+07 | 1.0E+06 | 100 | 1.1E+06 | 10 |
| 94 | 1-(4-Methoxyphenyl)-1,3-butanedi-one | 2.8E+05 | 1.2E+06 | 0.0E+00 | 93 | 4.5E+05 | 2.6E+06 | 0.0E+00 | 93 | 1.8E+05 | 38.7 |
| 115 | 4-Cyclopentene-1,3-dione | 4.2E+05 | 8.0E+05 | 1.8E+05 | 100 | 6.4E+05 | 5.4E+06 | 1.2E+05 | 100 | 2.2E+05 | 34.8 |
| 134 | Acetophenone | 2.3E+06 | 2.6E+07 | 2.5E+05 | 100 | 1.2E+06 | 3.6E+06 | 1.6E+05 | 100 | -1.1E+06 | -90.7 |
| 139 | 4-Hydroxy-4-methyl-5-hexenoic acid γ–lactone | 8.3E+05 | 3.5E+06 | 0.0E+00 | 81 | 7.9E+05 | 4.8E+06 | 0.0E+00 | 79 | -3.3E+04 | -4.1 |
| 154 | 1-H-Inden-1-one,2,3-dihydro-3,3,5,6-tetramethyl | 2.5E+07 | 8.1E+07 | 3.6E+06 | 100 | 1.3E+07 | 7.5E+07 | 6.8E+05 | 100 | -1.2E+07 | -90.5 |
| 158 | Cyclopenten-4-one, 1,2,3,3-tetrame-thyl- | 6.4E+05 | 1.8E+06 | 1.6E+05 | 100 | 3.2E+05 | 1.5E+06 | 0.0E+00 | 89 | -3.1E+05 | -97.9 |
| 163 | m-Methylacetophenone | 5.5E+06 | 2.6E+07 | 5.9E+05 | 100 | 4.7E+06 | 4.8E+07 | 3.4E+05 | 100 | -7.8E+05 | -16.7 |
| 166 | γ-Caprolactone | 2.8E+05 | 2.0E+06 | 0.0E+00 | 96 | 2.7E+05 | 1.6E+06 | 0.0E+00 | 89 | -2.1E+03 | -0.8 |
| 168 | β-Damascenone | 7.9E+06 | 2.2E+07 | 1.5E+06 | 100 | 5.8E+06 | 4.3E+07 | 1.1E+05 | 100 | -2.1E+06 | -37.3 |

| | | | | | | | | | | | |
|---|---|---|---|---|---|---|---|---|---|---|---|
| 182 | (E)-(2,4,4-Trimethylcyclohex-1,5-dien-1-yl]but-3-en-2-one | 9.3E+05 | 3.8E+06 | 0.0E+00 | 96 | 3.9E+05 | 2.5E+06 | 0.0E+00 | 96 | -5.4E+05 | -140.4 |
| 200 | 2,3,3-Trimethyl-2*H*-inden-1-one | 6.3E+05 | 1.9E+06 | 7.2E+04 | 100 | 2.0E+05 | 7.5E+05 | 0.0E+00 | 96 | -4.3E+05 | -215.8 |
| 210 | 4-(2,3,6-Trimethylphenyl)-2-butanone | 1.4E+06 | 6.7E+06 | 2.6E+05 | 100 | 8.8E+05 | 3.3E+06 | 4.3E+04 | 100 | -4.9E+05 | -55.8 |
| 216 | 1-(4-Hydroxy-3,5-di-tert.-butylphenyl)-2-methyl-3-morpholinopropan-1-one | 1.5E+07 | 8.6E+07 | 0.0E+00 | 96 | 3.8E+05 | 1.4E+06 | 0.0E+00 | 46 | -1.4E+07 | -3706.2 |
| | **TOTAL AREA** | **3.8E+08** | | | | **5.3E+08** | | | | | |

**Naphthalene derivatives**

| | | | | | | | | | | | |
|---|---|---|---|---|---|---|---|---|---|---|---|
| 73 | α-Ionene | 1.2E+07 | 3.6E+07 | 0.0E+00 | 93 | 6.2E+06 | 4.0E+07 | 0.0E+00 | 86 | -6.0E+06 | -97.6 |
| 93 | 2(1H)-Naphthalenone, 3,4,4a,5,6,7-hexahydro-1,1,4a-trimethyl- | 6.4E+06 | 3.6E+07 | 4.4E+05 | 100 | 1.1E+07 | 8.0E+07 | 0.0E+00 | 96 | 4.6E+06 | 41.6 |
| 156 | 1,2-Dihydro-1,1,6-trimethylnaphthalene | 8.2E+07 | 1.7E+08 | 1.2E+07 | 100 | 6.0E+07 | 1.8E+08 | 4.8E+06 | 100 | -2.2E+07 | -37.4 |
| 186 | 1,7-Dimethylnaphthalene | 3.7E+06 | 2.2E+07 | 4.0E+05 | 100 | 9.5E+05 | 4.4E+06 | 4.4E+04 | 100 | -2.8E+06 | -294.4 |
| 191 | 6-Hydroxy-3,8-dimethyl-5-propan-2-ylnaphthalene-1,2-dione | 7.2E+06 | 3.2E+07 | 4.0E+05 | 100 | 1.9E+06 | 8.4E+06 | 9.8E+04 | 100 | -5.2E+06 | -272.8 |
| 208 | Cadalene | 5.2E+07 | 1.9E+08 | 5.8E+06 | 100 | 2.0E+07 | 5.9E+07 | 1.1E+06 | 100 | -3.2E+07 | -160.9 |
| | **TOTAL** | **3.8E+08** | | | | **5.3E+08** | | | | | |

**Fatty Acids**

| | | | | | | | | | | | |
|---|---|---|---|---|---|---|---|---|---|---|---|
| 78 | Acetic acid | 6.7E+06 | 1.8E+07 | 7.5E+04 | 100 | 1.7E+07 | 1.3E+08 | 0.0E+00 | 96 | 1.0E+07 | 60.5 |
| 114 | Isobutyric acid | 4.3E+05 | 1.5E+06 | 1.4E+05 | 100 | 4.4E+05 | 3.2E+06 | 0.0E+00 | 93 | 1.0E+04 | 2.3 |

| | | | | | | | | | | | |
|---|---|---|---|---|---|---|---|---|---|---|---|
| 116 | 3-Pentenoic acid, 4-methyl- | 3.6E+05 | 1.2E+06 | 5.9E+04 | 100 | 4.4E+05 | 1.3E+06 | 2.9E+04 | 100 | 8.3E+04 | 18.7 |
| 141 | Ethylmethylacetic acid | 6.4E+05 | 2.0E+06 | 0.0E+00 | 93 | 1.2E+06 | 9.2E+06 | 0.0E+00 | 68 | 5.6E+05 | 46.8 |
| 192 | Caprylic acid | 7.6E+06 | 2.8E+07 | 1.3E+06 | 100 | 6.0E+06 | 2.5E+07 | 1.0E+06 | 100 | -1.5E+06 | -25.7 |
| 201 | Pelargonic acid | 1.8E+06 | 2.7E+06 | 6.2E+05 | 100 | 1.1E+06 | 3.3E+06 | 1.0E+05 | 100 | -6.2E+05 | -54.1 |
| 213 | Capric acid | 4.0E+06 | 9.4E+06 | 5.8E+05 | 100 | 2.4E+06 | 1.5E+07 | 9.0E+04 | 100 | -1.6E+06 | -65.4 |
| 219 | Dodecanoic acid | 1.3E+06 | 3.2E+06 | 2.8E+05 | 100 | 1.5E+06 | 8.8E+06 | 0.0E+00 | 89 | 1.6E+05 | 11.1 |
| | **TOTAL** | **2.3E+07** | | | | **3.0E+07** | | | | | |
| | **Organosulphurs** | | | | | | | | | | |
| 2 | Methanethiol | 1.2E+07 | 3.2E+07 | 3.3E+06 | 100 | 1.1E+07 | 4.4E+07 | 1.1E+06 | 100 | -1.6E+05 | -1.4 |
| 5 | 1-Propanethiol | 1.8E+05 | 1.0E+06 | 0.0E+00 | 85 | 7.4E+04 | 7.8E+05 | 0.0E+00 | 39 | -1.0E+05 | -138.6 |
| 24 | Dimethyldisulfide | 1.8E+08 | 4.3E+08 | 3.7E+07 | 100 | 3.2E+08 | 1.3E+09 | 4.1E+07 | 100 | 1.4E+08 | 43.0 |
| 42 | Methyl propyl disulfide | 5.8E+05 | 2.0E+06 | 1.0E+04 | 100 | 4.9E+05 | 3.8E+06 | 0.0E+00 | 89 | -9.7E+04 | -19.9 |
| 45 | 2,4-Dimethylthiophene | 3.2E+06 | 1.5E+07 | 0.0E+00 | 85 | 1.5E+06 | 9.3E+06 | 0.0E+00 | 75 | -1.7E+06 | -108.2 |
| 51 | Methyl 2-propenyl disulfide | 5.6E+06 | 3.3E+07 | 7.2E+04 | 100 | 2.7E+06 | 1.6E+07 | 1.8E+01 | 100 | -2.8E+06 | -103.4 |
| 97 | 3-(Methylthio)thiophene | 4.9E+05 | 1.8E+06 | 1.0E+05 | 100 | 3.8E+05 | 2.2E+06 | 2.6E+04 | 100 | -1.2E+05 | -30.5 |
| 143 | 2,5-Dimethylthiophene | 1.5E+06 | 3.7E+06 | 3.5E+05 | 100 | 1.4E+06 | 3.6E+06 | 3.1E+05 | 100 | -7.5E+04 | -5.3 |
| 178 | Dimethyl sulfone | 9.2E+05 | 2.1E+06 | 1.8E+05 | 100 | 7.5E+05 | 2.9E+06 | 0.0E+00 | 96 | -1.7E+05 | -22.6 |

| | | | | | | | | | | | |
|---|---|---|---|---|---|---|---|---|---|---|---|
| | **TOTAL** | **2.1E+08** | | | | **3.4E+08** | | | | | |
| | **Phenols** | | | | | | | | | | |
| 171 | 2-Chlorophenol | 3.1E+05 | 1.2E+06 | 4.7E+03 | 100 | 2.9E+05 | 1.7E+06 | 0.0E+00 | 96 | -1.5E+04 | -5.2 |
| 173 | Guaiacol | 3.5E+06 | 2.1E+07 | 9.2E+03 | 100 | 3.0E+06 | 1.8E+07 | 2.7E+04 | 100 | -5.0E+05 | -16.3 |
| 184 | 2-Bromophenol | 1.4E+05 | 2.6E+05 | 0.0E+00 | 96 | 2.9E+04 | 1.7E+05 | 0.0E+00 | 64 | -1.1E+05 | -389.4 |
| 188 | Phenol | 2.9E+07 | 9.1E+07 | 3.7E+02 | 100 | 5.6E+07 | 6.9E+08 | 0.0E+00 | 68 | 2.6E+07 | 47.6 |
| 195 | p-Cresol | 7.0E+07 | 3.6E+08 | 7.7E+05 | 100 | 2.5E+08 | 1.8E+09 | 7.5E+06 | 100 | 1.8E+08 | 72.6 |
| 196 | 2,4,6-Triisopropylphenol | 1.5E+05 | 5.6E+05 | 0.0E+00 | 93 | 6.0E+04 | 4.7E+05 | 0.0E+00 | 71 | -9.1E+04 | -152 |
| 197 | 4-tert-Butyl-2-chlorophenol | 1.9E+05 | 5.4E+05 | 0.0E+00 | 96 | 1.5E+05 | 4.0E+05 | 4.5E+04 | 100 | -3.5E+04 | -23.2 |
| 204 | 3-Hydroxybenzotrifluoride | 1.1E+06 | 8.1E+06 | 8.9E+04 | 100 | 1.9E+05 | 8.2E+05 | 0.0E+00 | 96 | -8.6E+05 | -451.2 |
| 205 | 5-Isopropyl-2-methylphenol | 8.9E+06 | 9.5E+07 | 4.9E+05 | 100 | 3.2E+06 | 1.7E+07 | 2.5E+05 | 100 | -5.7E+06 | -177.8 |
| 206 | Thymol | 1.4E+07 | 9.5E+07 | 1.3E+06 | 100 | 3.9E+06 | 2.5E+07 | 1.2E+05 | 100 | -1.1E+07 | -272.2 |
| 207 | 4-tert-Butyl-2-Bromophenol | 3.1E+05 | 7.1E+05 | 8.0E+04 | 100 | 1.2E+05 | 6.2E+05 | 1.1E+04 | 100 | -1.9E+05 | -155.3 |
| 214 | 4-tert-Butylphenol | 4.0E+05 | 9.4E+05 | 1.2E+05 | 100 | 3.9E+05 | 1.1E+06 | 1.2E+05 | 100 | -9.0E+03 | -2.3 |
| 215 | 2,4-Di-tert-butylphenol | 8.9E+07 | 1.9E+08 | 1.8E+07 | 100 | 6.1E+07 | 1.5E+08 | 6.7E+06 | 100 | -2.8E+07 | -45.6 |
| | **TOTAL** | **2.2E+08** | | | | **3.8E+08** | | | | | |
| | **Terpene Derivatives** | | | | | | | | | | |

| | | | | | | | | | | | |
|---|---|---|---|---|---|---|---|---|---|---|---|
| 34 | Limonene (isomer) | 3.3E+05 | 1.9E+06 | 4.5E+04 | 100 | 3.3E+05 | 1.6E+06 | 0.0E+00 | 89 | -4.7E+03 | -1.4 |
| 90 | α-Carene | 1.1E+06 | 8.7E+06 | 0.0E+00 | 93 | 7.7E+05 | 3.0E+06 | 0.0E+00 | 89 | -3.4E+05 | -44.3 |
| 92 | α-Ionone | 3.6E+06 | 1.3E+07 | 4.0E+05 | 100 | 4.5E+06 | 3.0E+07 | 4.9E+04 | 100 | 8.8E+05 | 19.8 |
| 95 | β-Ionone | 1.9E+06 | 8.3E+06 | 0.0E+00 | 96 | 2.2E+06 | 1.7E+07 | 0.0E+00 | 89 | 3.2E+05 | 14.1 |
| 100 | α-Cedrene | 2.2E+06 | 1.6E+07 | 1.6E+05 | 100 | 9.8E+05 | 6.7E+06 | 0.0E+00 | 96 | -1.2E+06 | -123.5 |
| 107 | Terpinenol | 5.8E+05 | 1.6E+06 | 9.4E+04 | 100 | 6.9E+05 | 4.8E+06 | 7.5E+04 | 100 | 1.1E+05 | 16.4 |
| 118 | (-)-Neoisomenthol | 3.7E+06 | 3.1E+07 | 0.0E+00 | 96 | 3.4E+06 | 4.4E+07 | 1.6E+04 | 100 | -2.9E+05 | -8.5 |
| 119 | *p*-Menth-1-en-4-ol | 4.2E+06 | 3.1E+07 | 0.0E+00 | 96 | 3.6E+06 | 4.8E+07 | 0.0E+00 | 89 | -5.8E+05 | -16.1 |
| 123 | Ylangene | 2.1E+06 | 1.6E+07 | 4.0E+05 | 100 | 1.0E+06 | 2.6E+06 | 2.4E+03 | 100 | -1.1E+06 | -106.1 |
| 124 | α-Caryophyllene | 1.9E+06 | 4.2E+06 | 1.9E+05 | 100 | 1.4E+06 | 9.8E+06 | 7.3E+04 | 100 | -5.1E+05 | -37.2 |
| 126 | Myrtenal | 5.6E+05 | 3.6E+06 | 1.5E+04 | 100 | 4.0E+05 | 2.6E+06 | 0.0E+00 | 96 | -1.7E+05 | -42.4 |
| 129 | Isoledene | 4.0E+06 | 3.1E+07 | 1.6E+04 | 100 | 1.2E+06 | 6.8E+06 | 0.0E+00 | 89 | -2.8E+06 | -235.1 |
| 130 | Menthol | 2.1E+07 | 7.7E+07 | 0.0E+00 | 96 | 6.1E+06 | 4.8E+07 | 0.0E+00 | 96 | -1.5E+07 | -246.9 |
| 131 | β-Chamigrene | 4.9E+06 | 1.6E+07 | 8.6E+05 | 100 | 2.9E+06 | 1.1E+07 | 0.0E+00 | 96 | -2.0E+06 | -71.0 |
| 132 | Viridiflorene | 2.3E+06 | 5.4E+06 | 4.2E+05 | 100 | 1.1E+06 | 5.6E+06 | 1.8E+05 | 100 | -1.2E+06 | -104.6 |
| 133 | β-Guaiene | 6.1E+06 | 1.5E+07 | 1.4E+06 | 100 | 3.2E+06 | 1.4E+07 | 2.1E+05 | 100 | -2.9E+06 | -90.1 |
| 135 | α-Amorphene | 6.2E+06 | 5.1E+07 | 0.0E+00 | 93 | 1.9E+06 | 1.1E+07 | 0.0E+00 | 89 | -4.3E+06 | -231.6 |

| | | | | | | | | | | | |
|---|---|---|---|---|---|---|---|---|---|---|---|
| 140 | (S)-Carvone | 1.1E+07 | 3.1E+07 | 1.4E+06 | 100 | 6.1E+06 | 3.6E+07 | 1.4E+05 | 100 | -5.2E+06 | -85.3 |
| 142 | 4,4-Dimethyl-3-(3-methylbut-3-enylidene)-2-methylenebicyclo[4.1.0]heptane | 2.2E+06 | 1.6E+07 | 2.0E+05 | 100 | 9.9E+05 | 4.3E+06 | 8.5E+04 | 100 | -1.2E+06 | -124.2 |
| 144 | α-Terpineol | 9.2E+04 | 3.0E+05 | 0.0E+00 | 89 | 1.5E+05 | 6.9E+05 | 0.0E+00 | 61 | 5.7E+04 | 38.1 |
| 146 | α-Muurolene | 1.4E+06 | 9.3E+06 | 6.6E+04 | 100 | 1.0E+06 | 6.6E+06 | 2.2E+04 | 100 | -4.4E+05 | -43.5 |
| 147 | β-Vatirenene | 1.9E+06 | 8.1E+06 | 1.7E+05 | 100 | 1.1E+06 | 4.7E+06 | 1.9E+04 | 100 | -8.3E+05 | -77.3 |
| 148 | α-Chamigrene | 6.1E+06 | 4.9E+07 | 0.0E+00 | 96 | 2.2E+06 | 9.7E+06 | 0.0E+00 | 96 | -3.9E+06 | -183.2 |
| 150 | Spathulenol | 2.1E+07 | 1.0E+08 | 1.9E+06 | 100 | 1.5E+07 | 8.5E+07 | 1.3E+05 | 100 | -5.3E+06 | -34.6 |
| 151 | Phellandral | 2.1E+06 | 1.4E+07 | 1.9E+05 | 100 | 9.4E+05 | 3.3E+06 | 1.5E+04 | 100 | -1.1E+06 | -119.6 |
| 161 | α-Curcumene | 1.4E+07 | 7.3E+07 | 9.2E+05 | 100 | 5.7E+06 | 4.0E+07 | 1.8E+04 | 100 | -7.9E+06 | -140.1 |
| 167 | 9-Isopropyl-1-methyl-2-methylene-5-oxatricyclo[5.4.0.0(3,8)]undecane | 3.8E+06 | 1.4E+07 | 1.7E+05 | 100 | 3.0E+06 | 3.4E+07 | 1.7E+04 | 100 | -7.9E+05 | -26.6 |
| 169 | Calamenene | 2.4E+07 | 1.3E+08 | 9.4E+05 | 100 | 1.4E+07 | 5.9E+07 | 3.5E+05 | 100 | -1.0E+07 | -73.7 |
| 179 | *α*-Calacorene | 1.3E+07 | 7.1E+07 | 4.8E+05 | 100 | 3.9E+06 | 2.2E+07 | 6.0E+04 | 100 | -9.1E+06 | -236.0 |
| 187 | Dehydro-β-Ionone | 6.9E+06 | 1.9E+07 | 4.4E+05 | 100 | 1.7E+06 | 5.9E+06 | 0.0E+00 | 89 | -5.2E+06 | -314.0 |
| 193 | γ-Muurolene | 5.8E+06 | 1.6E+07 | 5.1E+05 | 100 | 9.2E+06 | 7.1E+07 | 1.8E+05 | 100 | 3.4E+06 | 37.1 |
| 199 | (8*S*,8*aS*)-3,8-dimethyl-4-propan-2-ylidene-1,2,6,7,8,8*a*-hexahydroazulen-5-one | 8.8E+05 | 4.7E+06 | 4.7E+04 | 100 | 3.6E+05 | 1.7E+06 | 2.9E+04 | 100 | -5.2E+05 | -146.1 |

| | | | | | | | | | | | |
|---|---|---|---|---|---|---|---|---|---|---|---|
| 209 | 2,4a-Methanonaphthalen-7(4aH)-one, 1,2,3,4,5,6-hexahydro-1,1,5,5-tetramethyl-, (2s-cis)- | 8.2E+06 | 7.5E+07 | 3.4E+05 | 100 | 1.1E+07 | 6.6E+07 | 4.6E+04 | 100 | 2.6E+06 | 24.0 |
| 211 | ar-Turmerone | 4.2E+06 | 2.7E+07 | 4.8E+05 | 100 | 4.0E+06 | 2.2E+07 | 8.4E+04 | 100 | -2.7E+05 | -6.8 |
| | **TOTAL** | **1.9E+08** | | | | **1.2E+08** | | | | | |
| | **Other Compounds** | | | | | | | | | | |
| 1 | 1,2-Diaminopropane | 8.5E+07 | 2.6E+08 | 5.7E+07 | 100 | 8.6E+07 | 2.4E+08 | 2.2E+07 | 100 | 8.6E+05 | 1.0 |
| 23 | 3,4,5-Trimethylpyrazole | 4.5E+05 | 1.7E+06 | 2.6E+04 | 100 | 4.5E+05 | 1.7E+06 | 0.0E+00 | 93 | -2,1E+03 | -0.5 |
| 26 | Linaloyl oxide | 1.6E+05 | 5.4E+05 | 0.0E+00 | 93 | 5.2E+05 | 4.0E+06 | 0.0E+00 | 86 | 3,.6E+05 | 69.3 |
| 149 | 3,6-dimethyl-2H-indazole | 1.6E+06 | 6.3E+06 | 3.3E+05 | 100 | 7.2E+05 | 2.7E+06 | 0.0E+00 | 96 | -9.3E+05 | -129.3 |
| 217 | Benzoic acid | 4.8E+06 | 2.5E+07 | 8.7E+03 | 100 | 9.0E+06 | 9.8E+07 | 2.1E+05 | 100 | 8.6E+05 | 1.0 |
| | **TOTAL** | **8.7E+07** | | | | **8.7E+07** | | | | | |

**Table S2.** Results of the target groups discrimination following the use of different classification algorithms. The variables (uVOMs) used in each assay are described in Table 4.

| **All data processing** | | | | |
|---|---|---|---|---|
| ***Algorithm*** | ***Accuracy*** | ***Sensitivity (CTL)*** | ***Specificity (LC)*** | *AUC* |
| *SVM* | 96.33 | 92.59 | 100 | 0.975 |
| *RF* | 94.66 | 88.88 | 100 | 0.984 |
| *PLS-DA* | 96.33 | 96.29 | 96.42 | 0.989 |
| *MLP* | **98** | 96.29 | 100 | **0.999** |
| **Upon CFS variable selection** | | | | |
| *Algorithm* | *Accuracy* | *Sensitivity (CTL)* | *Specificity (LC)* | *AUC* |
| *SVM* | 96.31 | 92.59 | 100 | 0.996 |
| *RF* | 96.46 | 96.29 | 96.42 | 0.997 |
| *PLS-DA* | 96.31 | 92.59 | 100 | 0.998 |
| *MLP* | **97.83** | 97.40 | 98.21 | **0.999** |
| **Upon CFS + F-score variable selection** | | | | |
| *Algorithm* | *Accuracy* | *Sensitivity (CTL)* | *Specificity (LC)* | *AUC* |
| *SVM* | 100 | 100 | 100 | 1 |
| *RF* | 98.33 | 96.29 | 100 | 0.999 |
| *PLS-DA* | 96 | 92.59 | 100 | 0.997 |
| *MLP* | **100** | 97.40 | 98.21 | **0.999** |

**Table S3.** Pathway analysis results

| ***Pathway name*** | ***Total compounds*** | ***Match status*** | ***p*** | ***-log(p)*** | ***Holm p*** | ***FDR*** | ***Impact*** |
|---|---|---|---|---|---|---|---|
| Pyruvate metabolism | 22 | 1 | 0.057608 | 1.2395 | 0.28804 | 0.096014 | 0.06065 |
| Glycolysis / Gluconeogenesis | 26 | 1 | 0.057608 | 1.2395 | 0.28804 | 0.096014 | 0.02906 |
| Glyoxylate and dicarboxylate metabolism | 32 | 1 | 0.057608 | 1.2395 | 0.28804 | 0.096014 | 0 |
| Fatty acid biosynthesis | 47 | 3 | 0.23999 | 0.6198 | 0.47998 | 0.29999 | 0 |
| Sulfur metabolism | 8 | 1 | 0.95175 | 0.021475 | 0.95175 | 0.95175 | 0 |

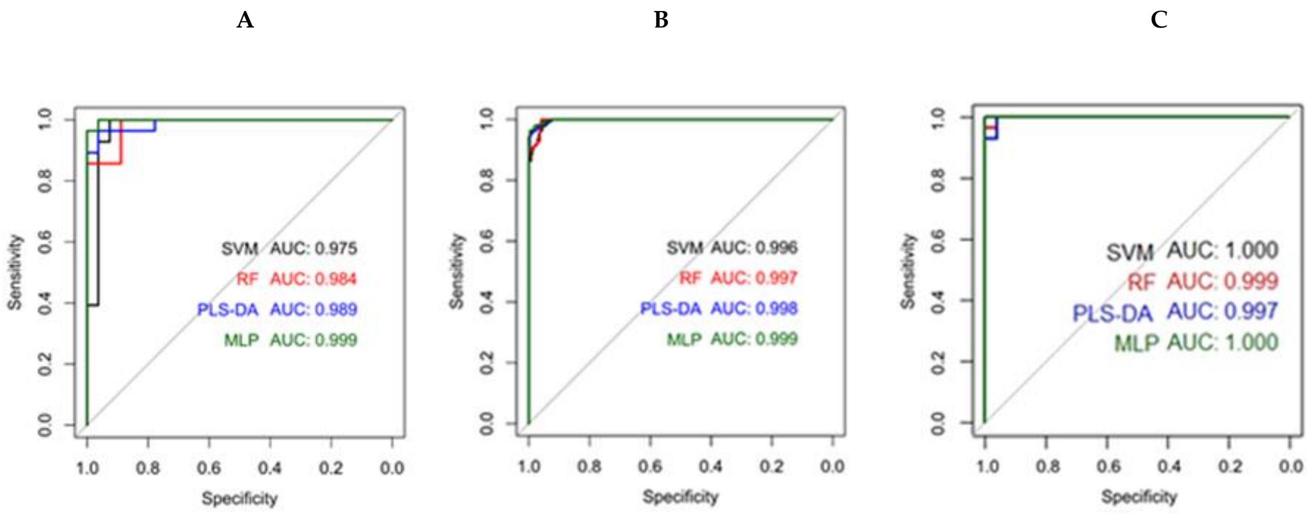


**Figure S1.** Performance of the variables identified with the different algorithms described in Table 2 and Table S3.

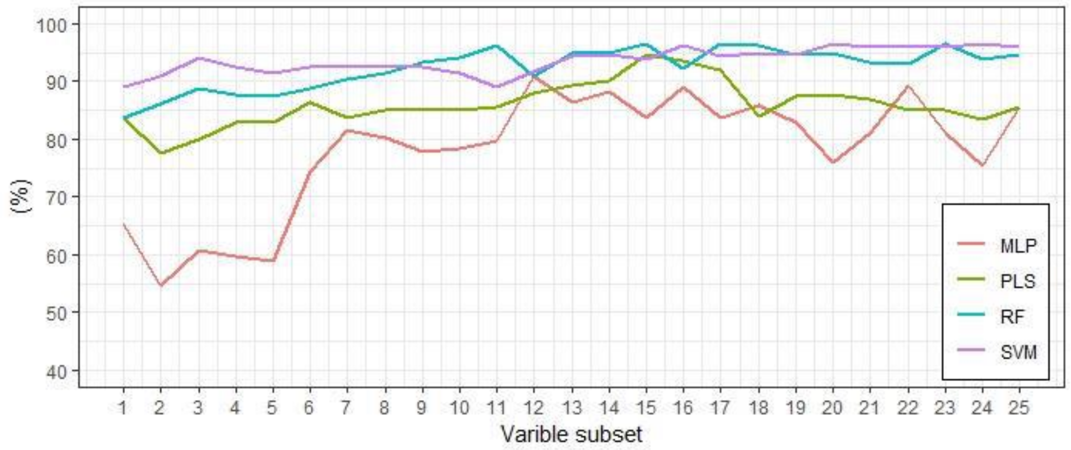


**Figure S2.** Results of the classification of the 25 groups generated from the F-score classification of the uVOMs identified in this work.
